# Relationship Between DWI-Based Acute Ischemic Stroke Volume, Location and Severity of Dysphagia

**DOI:** 10.3390/brainsci14121185

**Published:** 2024-11-26

**Authors:** Carlo A. Mallio, Daniele Vertulli, Gianfranco Di Gennaro, Maria Teresa Ascrizzi, Fioravante Capone, Chiara Grattarola, Vitaliana Luccarelli, Federico Greco, Bruno Beomonte Zobel, Vincenzo Di Lazzaro, Fabio Pilato

**Affiliations:** 1 Fondazione Policlinico Universitario Campus Bio-Medico, 00128 Rome, Italy; c.mallio@policlinicocampus.it (C.A.M.); daniele.vertulli@unicampus.it (D.V.); t.ascrizzi@policlinicocampus.it (M.T.A.); f.capone@policlinicocampus.it (F.C.); c.grattarola@policlinicocampus.it (C.G.); v.luccarelli@policlinicocampus.it (V.L.); b.zobel@policlinicocampus.it (B.B.Z.); v.dilazzaro@policlinicocampus.it (V.D.L.); 2 Research Unit of Radiology, Department of Medicine and Surgery, Università Campus Bio-Medico di Roma, 00128 Rome, Italy; federico.greco@unicampus.it; 3 Department of Health Sciences, Chair of Medical Statistics, University of Catanzaro “Magna Græcia”, 88100 Catanzaro, Italy; gianfranco.digennaro@unicz.it; 4 Research Unit of Neurology, Fondazione Policlinico Universitario Campus Bio-Medico, 00128 Rome, Italy; 5 Research Unit of Otorhinolaryngology (ENT), Department of Medicine and Surgery, Università Campus Bio-Medico di Roma, 00128 Rome, Italy; 6 Department of Radiology, Cittadella della Salute, Azienda Sanitaria Locale di Lecce, Piazza Filippo Bottazzi, 73100 Lecce, Italy

**Keywords:** brain, MRI, stroke, DWI, dysphagia

## Abstract

Background/Objectives: The impact of stroke location and volume on the development of post-stroke dysphagia is not fully understood. The aim of this study is to evaluate the relationship between acute ischemic lesions and the severity of dysphagia. Methods: Brain MRIs were obtained with a 1.5 Tesla MRI system (Magnetom Avanto B13, Siemens, Erlangen, Germany). The brain MRI protocol included axial echo planar diffusion-weighted imaging (DWI). The acute ischemic volume was obtained using DWI by drawing regions of interest (ROIs). The diagnosis and assessment of the severity of dysphagia was carried out by a multidisciplinary team and included the Dysphagia Outcome and Severity Scale (DOSS), the Penetration–Aspiration Scale (PAS), and the Pooling score (P-score). The threshold for statistical significance was set at 5%. Results: Among all the patients enrolled (*n* = 64), 28 (43.8%) were males and 36 (56.2%) were females, with a mean age of 78.8 years. Thirty-three (51.6%) of them had mild dysphagia and thirty-one (48.4%) had moderate–severe dysphagia. The total ischemic volume was negatively correlated with the DOSS (r = −0.441, *p* = 0.0003) and positively with the P-score (r_s_ = 0.3054, *p* = 0.0328). Conclusions: There are significant associations between the severity of dysphagia and the quantitative DWI-based data of the acute ischemic volume and anatomical location.

## 1. Introduction

As a complex sensorimotor action, swallowing requires coordinated brain activity at the cortical and subcortical levels. Dysphagia, defined as swallowing impairment, can develop after injuries to this complex multi-level network. Acute ischemic stroke patients often experience dysphagia, with a frequency of about 78% [1]. Around two-thirds of patients develop acute dysphagia then experience aspiration [2]. This is finally linked to a higher incidence of hospital complications, such as malnutrition and aspiration pneumonia, as well as a worse prognosis for long-term functional outcomes [3,4]. Thus, the early identification and management of dysphagia are essential for improving outcomes in stroke patients. To correctly manage the patient’s nutrition status, drug administration, and additional instrumental diagnostics, such as a fiberoptic endoscopic evaluation of swallowing and/or a videofluoroscopic swallowing study, is crucial to identify patients with post-stroke dysphagia as soon as possible. Therefore, a routine dysphagia screening is a requirement of current stroke care for all patients [5,6,7].

In this context, recent research has focused on early biomarkers and neuroimaging to predict dysphagia recovery, helping to reduce complications and improve long-term outcomes.

Diffusion-weighted imaging (DWI) is a crucial imaging technique in the field of neurology and radiology, which is used to diagnose and assess ischemic stroke. DWI measures the movement of water molecules in brain tissue, and during an ischemic stroke, restricted diffusions of water occurring due to reduced blood flow are visualized as a hyperintense signal in DWI images and a low signal intensity in apparent diffusion coefficient (ADC) maps [8,9]. This imaging technique is particularly helpful to quickly identify the location and extent of the acute ischemic stroke.

DWI’s ability to precisely identify stroke-affected areas has made it invaluable in assessing regions likely to impact swallowing function, particularly by localizing damage to cortical and subcortical networks involved in sensorimotor control [10,11,12]. Integrating DWI findings with clinical assessments allows for an earlier prognosis of dysphagia and targeted therapeutic interventions, potentially mitigating risks like aspiration and malnutrition. As research advances, combining DWI with other imaging biomarkers could refine the prediction models [13], possibly enabling personalized dysphagia management and improving post-stroke recovery outcomes.

This study aims to investigate the relationship between the location and volume of acute ischemic lesions, as determined through DWI segmentation, and the severity of dysphagia in stroke patients. Moreover, this study hypothesizes that the acute ischemic stroke volume and location might be significantly associated with dysphagia severity, with specific brain regions possibly involved according to their distinct roles in swallowing impairment due to their motor and sensory functions.

By mapping and quantifying lesion characteristics, we seek to understand how specific regions and the extent of ischemic damage impact swallowing function. This approach could provide insights into which brain areas are most critical for preserving or recovering swallowing abilities and help in predicting dysphagia severity and progression.

## 2. Materials and Methods

The study was approved by the Ethical Committee of the Fondazione Policlinico Universitario Campus Bio-Medico, on the date of 9 September 2022 (N. PROT.: 57.22). All the methods adhered to the Declaration of Helsinki’s ethical principles, and all participants provided their signed informed permission. The study population included 64 patients admitted to the Stroke Unit Neurology Department of our hospital.

The inclusion criteria for this study were as follows: (1) patients aged over 18 years; (2) a confirmed diagnosis of mild to severe dysphagia; and (3) the availability of magnetic resonance imaging (MRI) obtained during hospitalization with a confirmed diagnosis of acute ischemic stroke.

The exclusion criteria were designed to ensure the reliability and precision of the findings and included (1) the presence of pre-existing neurological or psychiatric disorders that could confound dysphagia assessment; (2) vascular, neoplastic, or traumatic lesions of the central nervous system unrelated to acute ischemic stroke; (3) a history of brain irradiation or neurosurgery, which might alter the brain structure or function; (4) absolute contraindications to MRI (e.g., pacemakers or other metallic implants); and (5) poor-quality MRI images caused by artifacts, which could compromise the accurate assessment of the ischemic volume and location.

The diagnosis and assessment of the severity (i.e., mild vs. moderate–severe) of dysphagia was carried out by a multidisciplinary team, including the application of three scales: the Dysphagia Outcome and Severity Scale (DOSS), the Penetration–Aspiration Scale (PAS), and the Pooling score (P-score). The diagnosis of dysphagia was established within one week after the execution of brain MRI.

Acute phase therapy was administered based on hospital arrival times, in strict accordance with current guidelines [14]. All patients subsequently received secondary prevention therapy, consisting of either antiplatelet or anticoagulant treatment, depending on specific clinical indications.

### 2.1. Functional Evaluation of Dysphagia

The DOSS is a 7-point scale that is straightforward and easy to use [15]. It was created to objectively measure the functional severity of dysphagia based on the three factors that were most closely related to the final recommendations (i.e., oral stage bolus transfer, pharyngeal stage retention, and airway protection). The scale ranges from 7 (within normal bounds) to 3 (aspiration of water), 2 (aspiration of food), and 1 (aspiration of saliva).

The P-score is used as a scale for the endoscopic clinical evaluation of the dysphagia severity by evaluating the stagnation in the hypopharynx and larynx [16]. It considers a number of factors, including (1) the site, which may be determined by anatomical markers; (2) the quantity, as assessed semi-quantitatively by the amount of pooled materials (the coating, greater or less than 50% of the cavity containment capacity); and (3) management, as assessed by the patient’s capacity to expel the residual. Such an assessment is regarded as a clinical severity criterion that might be connected to the possibility of respiratory impairment. It goes from 4 to 11.

The PAS allows us to define the degree of severity of aspiration and penetration observed during the videofluoroscopic evaluation [17]. With 1 denoting the least severe score and 8 the highest or most severe, it is an 8-point ordinal scale. PAS scores are multidimensional, i.e., each score contains a number of observations: (1) the degree of airway invasion (i.e., whether the material is above, touching, or below the level of the vocal folds); (2) whether the material is expelled or not after swallowing; and (3) the patient’s reaction to the presence of the material in the airway (i.e., effort to remove the material).

### 2.2. MRI Parameters

The brain MRIs of patients were obtained using a 1.5 Tesla MRI system (Magnetom Avanto B13, Siemens, Erlangen, Germany). The brain MRI protocol included axial echo planar DWI (TR, 3927 msec; TE, 106 msec; matrix, 128 × 128; FOV, 23 × 23 cm; slice thickness, 5 mm) with a diffusion sensitivity [b] = 0 and three orthogonal diffusion-encoding gradients with b = 1000 [8,18,19].

**Figure 1:**
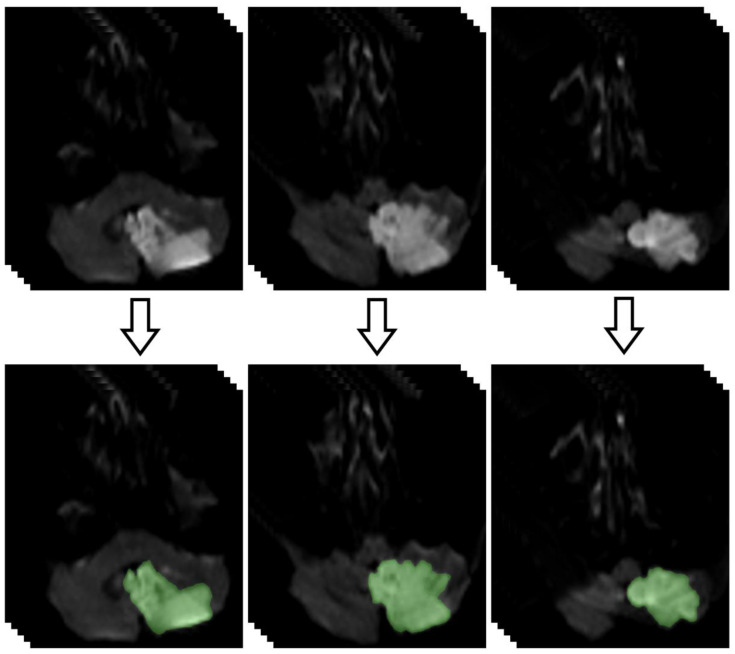
DWI images showing the segmentation of a left cerebellar acute stroke to calculate the total volume of the lesion.

Measurements of the brain ischemic volume were obtained using DWI as follows. First, the regions of interest (ROIs) of the ischemic area (cm^2^) were segmented and calculated for each subject using a function of the OsiriX MD v.2.6 software. Then, area values were multiplied by the thickness of the image slice to obtain values of the ischemic volume (cm^3^) (Figure 1).

The values of the ischemic volume were collected separately according to the anatomic location (the brainstem, frontal lobe, parietal lobe, temporal lobe, occipital lobe, hemi-cerebellum, and basal ganglia, both right and left) and brain vascularization territories anterior cerebral artery (ACA), middle cerebral artery (MCA), posterior cerebral artery (PCA), and vertebrobasilar arteries. An experienced neuroradiologist (C.A.M., 13 years of experience) visually inspected all the segmentations and verified the accuracy of the measurements [20].

### 2.3. Statistical Analysis

Since most of the variables exhibited a skewed distribution, the median and interquartile range were used to describe ischemic volumes obtained in different locations and brain vascular territories. The Wilcoxon–Mann–Whitney test was used to assess differences between mild and moderate–severe dysphagia.

Correlations between all the ischemic volume locations and dysphagia degrees (using the DOSS, P-score, and PAS) were evaluated with the nonparametric Spearman correlation coefficient, and the corresponding *p* values were calculated.

The significance level was set at 5%. The statistical analyses were performed using STATA (version 17.0, StataCorp, College Station, TX, USA, www.stata.com, accessed on 24 November 2024).

## 3. Results

Among all the patients enrolled (*n* = 64), 28 (43.8%) were males and 36 (56.2%) were females, with a mean age of 78.8 years (a range of 51–93 years). Six patients over 64 (9.4%) received an acute treatment with recombinant tissue plasminogen activator (r-tPA). Thirty-three (51.6%) over 64 patients had mild dysphagia and thirty-one (48.4%) had moderate–severe dysphagia. The median total ischemic volume was 2.08 (IQR: 0.38, 12.28) cm^3^; considering the location, the median ischemic volume was 1.32 (IQR: 0.38, 8.83) cm^3^ in the right supratentorial region, 4.56 (IQR: 0.30, 16.88) cm^3^ in the left supratentorial region, 0.94 (IQR: 0.43, 2.11) cm^3^ in the cerebellum, and 0.27 (IQR: 0.13, 0.38) cm^3^ in the brainstem. Considering the vascular anatomic territories, the median ischemic volume was 2.62 (IQR: 0.38, 14.39) cm^3^ in the anterior circulation and 0.51 (IQR: 0.37, 1.55) cm^3^ in the posterior circulation. The ischemic volumes were left supratentorial in 31 patients (43%), right supratentorial in 27 patients (38%), cerebellar in 8 patients (11%) and in the brainstem of 6 patients (8%). The ischemic lesions were of the anterior circulation in 56 cases and of the posterior circulation in 14 cases.

No statistically significant differences in the total ischemic volume, anatomical regions, and vascular regions were found when comparing subjects with mild and moderate–severe dysphagia.

The overall and dysphagia severity-stratified median values and IQR are reported in Table 1, along with the significance of the Wilcoxon–Mann–Whitney test.

**Figure 2:**
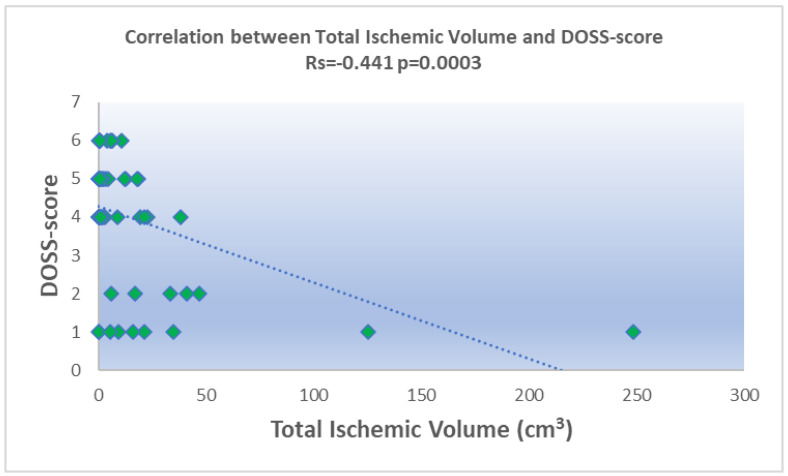
Scatterplot showing the statistically significant negative correlation between the Dysphagia Outcome and Severity Scale (DOSS) and the total acute ischemic volume.

**Figure 3:**
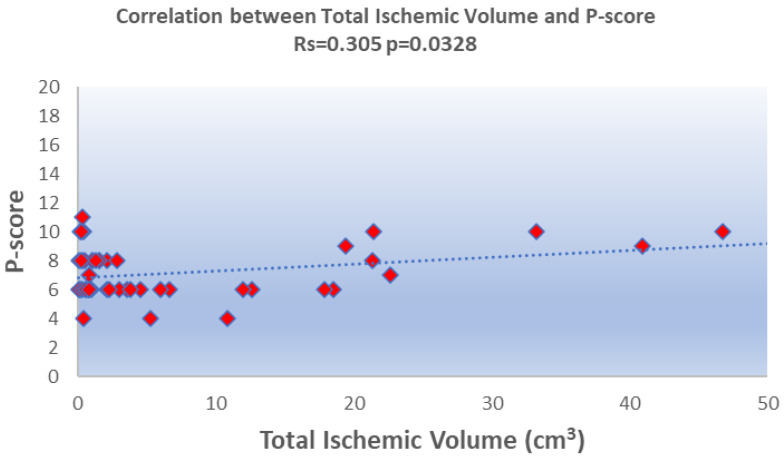
Scatterplot showing the statistically significant positive correlation between the Pooling score (P-score) and the total acute ischemic volume.

The total ischemic volume was negatively correlated with the DOSS (r = −0.441, *p* = 0.0003) and positively with the P-score (r_s_ = 0.3054, *p* = 0.0328) (Figure 2 and Figure 3). No significant correlation was found with the PAS.

Individual brain regions showing correlations with the DOSS were both right and left supratentorial (r_s_ = −0.5231 and *p* = 0.0051 and r_s_ = −0.4208 and *p* = 0.0184, respectively), right parietal (r_s_ = −0.605, *p* = 0.0488), and the left basal ganglia (r_s_ = −0.581, *p* = 0.0372). Moreover, the same scale was found to be correlated with the cerebellum (r_s_ = −0.8495, *p* = 0.0076) and left cerebellum (r_s_ = −0.999, *p* = 0.0001). The only brain location which was correlated with the P-score was the cerebellum (r_s_ = 0.884, *p* = 0.0036).

Considering vascular territories, the DOSS was also correlated with both the anterior and posterior brain circulation (r_s_ = −0.471 and *p* = 0.0002 and r_s_ = −0.577 and *p* = 0.0389, respectively) and with the right MCA (r_s_ = −0.582, *p* = 0.0028), left MCA (r_s_ = −0.363, *p* = 0.0485), and left vertebrobasilar (r_s_ = −0.9999, *p* = 0.0001) territories. The only vascular brain territory correlated with the P-score was the anterior circulation (r_s_ = 0.320, *p* = 0.0412). For details, see Table 2.

## 4. Discussion

In this study, we identified statistically significant correlations between acute ischemic volumes, as measured through DWI, and dysphagia severity as assessed by the DOSS and P-score scale, whereas no significant correlation was found with the PAS score. Specifically, larger ischemic volumes were associated with a decrease in DOSS scores and an increase in P-scores, both indicating a worsening swallowing impairment as the ischemic volume expands. This trend suggests that the extent of ischemic damage plays a central role in dysphagia severity, with the total ischemic volume emerging as a valuable indicator for clinical assessments.

An analysis of specific anatomic regions further clarified these findings. Ischemia in the cerebellum correlated significantly with both the DOSS and P-scores, highlighting the cerebellum’s potential role as a key region in predicting dysphagia severity. Thus, the cerebellum, crucial for motor control, can be linked to dysphagia when affected by ischemia. This association aligns with the cerebellum’s involvement in coordinating fine motor control and sensorimotor integration, which are essential for swallowing function [21,22]. Indeed, it integrates sensory feedback and coordinates the precise motor actions required for swallowing. Cerebellar damage could disrupt these processes, impairing timing, coordination, and reflex modulation [21,22]. Additionally, its connections to brainstem and cortical swallowing centers may be compromised, further affecting swallowing function.

In addition, ischemic volumes in the right parietal cortex and left basal ganglia were correlated with DOSS scores alone, emphasizing the impact of both cortical and subcortical structures on dysphagia. These findings suggest that damage within both cortical regions, such as the right parietal lobe, and subcortical structures, like the left basal ganglia, can contribute to swallowing impairment, albeit with varying degrees of impact depending on the lesion’s specific location. The right parietal lobe supports sensorimotor integration and adapts swallowing to sensory input, while the left basal ganglia coordinate motor planning and timing for swallowing. Thus, ischemia in these regions might affect distinct aspects of swallowing.

Collectively, these correlations highlight the value of lesion mapping in predicting dysphagia severity, offering potential clinical applications for targeted imaging-based assessments. By identifying key regions that affect swallowing function, clinicians may better predict and address the risks of dysphagia following ischemic stroke, tailoring interventions according to the volume and location of ischemic lesions. These findings highlight the potential of lesion-specific analyses to enhance dysphagia prediction and management in stroke patients.

In short, the observed findings underscore the role of the cerebellum, right parietal cortex, and left basal ganglia in sensorimotor integration relevant to swallowing, underscoring the impact of both cortical and subcortical networks on swallowing function and highlighting these potential predictive biomarkers for dysphagia severity [10,11,12,21,22]. This anatomical specificity in dysphagia severity markers suggests that the targeted neuroimaging of these regions could improve early prognostication and allow for more personalized interventions [5,6,7].

Moreover, the ischemic areas located in the anterior brain circulation showed a statistically significant relationship with both the DOSS and P-score, especially in both right and left MCA territories, while left vertebrobasilar ischemia were correlated only with the DOSS.

These findings suggest that ischemic lesions in the anterior brain circulation, particularly in the MCA territory, play a critical role in dysphagia severity [11,12]. Lesions in the MCA might be linked to dysphagia due to its supply to key swallowing pathways. These include the primary motor and sensory cortices, which control and process movements of the face and pharynx; the insular cortex, crucial for swallowing initiation and modulation; and the basal ganglia, which support motor coordination [11,12]. The involvement of both right and left MCA regions highlights the bilateral importance of these areas in coordinating swallowing, likely due to their roles in sensorimotor processing.

The significant association between left vertebrobasilar ischemia and DOSS scores, despite their lack of a correlation with the P-score, underscores the nuanced impact of the posterior circulation on swallowing, potentially affecting more localized motor control aspects.

Taken together, these results indicate that ischemic events in both the anterior and posterior circulations contribute differently to dysphagia severity, with anterior lesions broadly impacting the overall function and posterior lesions influencing specific motor control [23].

Our results are consistent with the literature on the topic. Lapa et al. [24] studied the possibility of correlating various ischemic lesion types with dysphagia using the Alberta stroke program early CT score (ASPECTS). The lentiform nucleus, the insula, and the frontal operculum were shown to have the highest relationships with dysphagia in left hemisphere strokes (OR of 0.113, 0.275, and 0.280). Combining two of these areas, or even all three, the relative dysphagia frequency increased by 100%. Only non-significant relationships were discovered for right hemispheric strokes, with the insula area showing the highest correlation.

It is well established that the insular cortex and the inferior pre-central gyrus serve as important nodes in the supratentorial deglutition network [25]. Numerous investigations have consistently shown that these regions are generally involved in swallowing; however, the results are non-uniform about lateralization to the right or left hemisphere [26]. The left insula is known to process sensory information and interacts with several brain areas across both hemispheres to play a significant role in the swallowing reflex [27]. Additionally, neuroimaging studies have supported the concept that the lentiform nucleus participates in swallowing, despite the paucity of information on its deglutition functions as a component of the basal ganglia [28].

Recently, Mihai et al. [29] applied functional magnetic resonance imaging (fMRI) and DWI studies to obtain insights into the swallowing of 18 individuals who had undergone stroke and had recovered from dysphagia. The laterality index of fractional anisotropy was extremely asymmetric and inversely correlated with the lesion size, with white matter lesions of the pyramidal tract between the M1/S1 tongue region and the posterior limb of the internal capsule being the most relevant. They concluded that the lesion location, rather than the size alone, plays a more important role in swallowing disorders, and this conclusion can also partly be applied to our results.

The relationship between the ischemic volume and dysphagia severity could be influenced by several key factors. The patient age could affect recovery due to reduced neural plasticity and common comorbidities, while the stroke type and lesion location (e.g., the cerebellum, basal ganglia, MCA territory) play crucial roles due to their impact on motor and sensorimotor functions. Pre-existing comorbidities like diabetes or neurodegenerative diseases, as well as the baseline swallowing status, can exacerbate impairments. Additionally, the timing and quality of interventions might affect outcomes. Addressing these variables is essential for accurate assessment and personalized management.

Future directions indicate that artificial intelligence (AI) could significantly improve dysphagia prediction and management post-stroke [30,31,32]. Machine learning models trained on neuroimaging data, such as DWI and fMRI, can identify complex patterns linking lesion location and dysphagia severity, possibly enhancing predictive accuracy. By integrating real-time analysis, AI could pinpoint high-risk brain regions, aiding personalized treatment approaches. As datasets grow, AI-driven models may further refine predictions, enabling targeted rehabilitation and better outcomes for stroke patients [30,31,32]. On this respect, deep learning algorithms based on artificial neural networks could be applied to process and integrate structural and functional complex brain MRI data [33].

Indeed, knowing that a stroke affects high-risk areas for dysphagia might enable early screening, nutritional adjustments, and targeted swallowing therapy to prevent complications like aspiration pneumonia. Mechanistic insights, such as the role of specific regions (e.g., the cerebellum, basal ganglia, MCA territories), could help to refine personalized interventions, such as specific rehabilitation and nutrition strategies, possibly improving recovery predictions.

The limitations of the present study should be taken into account when interpreting our findings. First, the retrospective design inherently limits the ability to establish causality, as it relies on previously collected data, which may lack the detailed controls of a prospective study. Additionally, the relatively small sample size may reduce the generalizability of the results, as the limited number of patients may not fully represent the diverse presentations and severities of dysphagia in the broader population of stroke patients. This limitation is particularly relevant for less common ischemic lesions, such as those in the brainstem, which were underrepresented in our sample despite their known importance in swallowing function. Indeed, it would be interesting to explore the relationship between structural and functional brain connectivity in patients experiencing dysphagia after ischemic stroke [34,35].

Hemispheric laterality should be further evaluated as it might influence dysphagia severity. Although the ischemic volume impacts dysphagia across both hemispheres, larger lesions may overshadow lateralized differences. Additionally, the potential synergistic effects of lesions located in multiple regions on dysphagia severity deserve to be further explored. Understanding these effects can further improve targeted interventions based on the lesion location.

Future studies on larger series should be carried out to confirm our data and shed light on the complex link between stroke and dysphagia.

## 5. Conclusions

This study demonstrates significant associations between dysphagia severity and quantitative DWI-based data, specifically the ischemic lesion volume and anatomical location in acute ischemic stroke patients. The correlation between lesion characteristics and dysphagia severity provides new insights into the underlying mechanisms of post-stroke swallowing impairment. These findings underscore the importance of targeted neuroimaging in identifying high-risk patients and predicting dysphagia severity, particularly with lesions in regions like the cerebellum, basal ganglia, and MCA territories, which are crucial for swallowing function. The future integration of DWI-based insights with other neuroimaging and clinical assessments may enhance predictive accuracy and inform personalized rehabilitation plans, ultimately improving outcomes for patients with post-stroke dysphagia.

## Figures and Tables

**Table 1:** Descriptive analysis of overall, mild, and moderate–severe dysphagia.

	Overall			Mild Dysphagia			Moderate–Severe Dysphagia			*p*
	Median	IQR		Median	IQR		Median	IQR		
Total ischemic volume	2.08	0.38	12.28	1.55	0.54	6.01	2.81	0.30	21.34	0.32
Right frontal lobe	0.51	0.21	4.80	1.37	0.38	2.24	0.21	0.12	10.10	0.53
Right parietal lobe	3.83	1.06	6.00	3.43	2.57	4.92	4.03	0.56	32.89	1
Right occipital lobe	1.00	0.16	21	0.06	0.06	0.06	11	0.58	30.96	0.40
Right temporal lobe	0.46	0.20	16.34	8.27	0.20	16.34	0.46	0.33	13.89	1
Right basal ganglia	0.54	0.22	1.56	0.55	0.31	3	0.19	0.16	0.22	0.22
Right cerbellum	1.05	0.83	1.55	1.19	0.83	1.55	1.05	0.16	2.67	1
Left frontal lobe	4.46	0.26	11.13	6.14	0.65	9.67	2.96	0.16	13.20	0.64
Left parietal lobe	3.23	1.07	17.49	2.09	1.78	4.56	4.37	0.16	21.34	0.56
Left occipital lobe	1.28	0.16	3.20	0.79	0.30	1.28	1.66	0.14	18.10	0.89
Left temporal lobe	5.31	1.45	15	3.54	3.54	3.54	7.08	1.45	15	1
Left basal ganglia	0.37	0.23	0.85	0.50	0.37	0.59	0.26	0.16	1.59	0.52
Left cerebellum	0.35	0.2	10.94	0.43	0.43	0.43	0.27	0.13	21.45	1
Brainstem	0.27	0.13	0.38	1.13	0.16	2.09	0.25	0.09	0.38	0.53
Right ACA	24.39	16.18	32.60				24.39	16.18	32.60	
Right MCA	1.19	0.42	5.63	2.24	0.38	5.25	1.06	0.46	8.83	1
Right PCA	0.15	0.06	40.91	0.06	0.06	0.06	20.53	0.15	40.91	0.67
Right vertebrobasilar	0.61	0.16	1.55	1.19	0.50	1.82	0.31	0.13	1.05	0.48
Left ACA	0.73	0.20	1.27	0.20	0.20	0.20	1.27	1.27	1.27	1
Left MCA	4.42	0.37	12.60	4.56	0.60	11.97	4.35	0.23	19.39	0.73
Left PCA	6.61	0.15	21.40				6.61	0.15	21.40	
Left vertebrobasilar	0.34	0.19	10.94	0.43	0.43	0.43	0.24	0.13	21.45	1
Right sovra-tentorial	1.32	0.38	8.83	1.48	0.37	4.54	1.32	0.46	40.91	0.37
Left sovra-tentorial	4.56	0.30	16.88	4.56	0.80	11.97	3.35	0.19	20.36	0.73
Cerebellum	0.94	0.43	2.11	0.83	0.43	1.55	1.05	0.43	2.67	1
Brainstem	0.27	0.13	0.38	1.13	0.16	2.09	0.25	0.09	0.38	0.53
Anterior circulation	2.62	0.38	14.39	2.24	0.54	6.60	5.62	0.28	21.34	0.40
Posterior circulation	0.51	0.37	1.55	0.83	0.43	1.55	0.47	0.25	1.86	0.72

**Table 2:** Pearson’s correlation analysis between brain ischemic volume and DOSS, P-score, and PAS. The asterisk means statistically significant results.

	DOSS		P-Score		PAS	
	r	*p*	r	*p*	r	*p*
Total	−0.441	0.0003 *	0.305	0.0328 *	−0.048	0.7481
Right frontal lobe	−0.504	0.0789	−0.190	0.6527	−0.509	0.1979
Right parietal lobe	−0.605	0.0488 *	0.560	0.1492	0.296	0.4771
Right occipital lobe	−0.450	0.4469	0.757	0.453	−0.988	0.1008
Right temporal lobe	−0.595	0.1589	0.089	0.867	−0.131	0.8043
Right basal ganglia	0.203	0.6001	−0.532	0.2195	−0.488	0.2666
Right cerebellum	−0.162	0.7944	0.252	0.6821	−0.112	0.8578
Left frontal lobe	−0.067	0.8066	−0.332	0.3194	−0.384	0.2435
Left parietal lobe	−0.320	0.2271	0.175	0.6071	0.258	0.4438
Left occipital lobe	−0.456	0.2177	0.517	0.2937	−0.891	0.1089
Left temporal lobe	−0.500	0.3127	0.737	0.4725	−1	1
Left basal ganglia	−0.581	0.0372 *	−0.425	0.2212	−0.096	0.8062
Left cerebellum	−0.999	0.0001 *	0.923	0.0768	−0.946	0.0538
Brainstem	0.293	0.573	−0.463	0.3556	−0.701	0.1868
Right ACA						
Right MCA	−0.582	0.0028 *	0.328	0.1837	0.115	0.6483
Right PCA	−0.946	0.2111	0.757	0.4532	−0.988	0.1006
Right vertebrobasilar	0.153	0.6735	−0.109	0.7647	−0.345	0.3283
Left ACA	−1	1				
Left MCA	−0.363	0.0485 *	−0.043	0.849	−0.074	0.7495
Left PCA	−0.734	0.0965	1	0.0001 *	1	0
Left vertebrobasilar	−0.999	0.0001 *	0.923	0.077	−0.946	0.0536
Right supratentorial	−0.523	0.0051 *	0.443	0.0504	−0.036	0.8802
Left supratentorial	−0.421	0.0184 *	0.110	0.6166	−0.076	0.7356
Cerebellum	−0.850	0.0076 *	0.884	0.0036 *	−0.226	0.5908
Brainstem	0.293	0.573	−0.463	0.3556	−0.701	0.1868
Anterior circulation	−0.471	0.0002 *	0.320	0.0412 *	−0.037	0.8194
Posterior circulation	−0.577	0.0389 *	0.509	0.0758	−0.185	0.565

## Data Availability

The raw data supporting the conclusions of this article will be made available by the authors on request.

## Abbreviations

ACA	anterior cerebral artery
ADC	apparent diffusion coefficient
ASPECTS	Alberta stroke program early CT score
DOSS	Dysphagia Outcome and Severity Scale
DWI	diffusion-weighted imaging
FEES	fiberoptic endoscopic assessment of swallowing
MCA	middle cerebral artery
MRI	magnetic resonance imaging
PAS	Penetration–Aspiration Scale
PCA	posterior cerebral artery
P-score	Pooling score
ROIs	regions of interest
